# Akt inhibitors as an HIV-1 infected macrophage-specific anti-viral therapy

**DOI:** 10.1186/1742-4690-5-11

**Published:** 2008-01-31

**Authors:** Pauline Chugh, Birgit Bradel-Tretheway, Carlos MR Monteiro-Filho, Vicente Planelles, Sanjay B Maggirwar, Stephen Dewhurst, Baek Kim

**Affiliations:** 1 Department of Microbiology and Immunology, School of Medicine, University of Rochester Medical Center 601 Elmwood Avenue Box 672 Rochester, New York 14742 USA; 2 Division of Cellular Biology and Immunology, Department of Pathology, University of Utah School of Medicine, 30 N 1900 East, SOM 5C210, Salt Lake City, UT 84132. USA

## Abstract

**Background:**

Unlike CD4+ T cells, HIV-1 infected macrophages exhibit extended life span even upon stress, consistent with their *in vivo *role as long-lived HIV-1 reservoirs.

**Results:**

Here, we demonstrate that PI3K/Akt inhibitors, including clinically available Miltefosine, dramatically reduced HIV-1 production from long-living virus-infected macrophages. These PI3K/Akt inhibitors hyper-sensitize infected macrophages to extracellular stresses that they are normally exposed to, and eventually lead to cell death of infected macrophages without harming uninfected cells. Based on the data from these Akt inhibitors, we were able to further investigate how HIV-1 infection utilizes the PI3K/Akt pathway to establish the cytoprotective effect of HIV-1 infection, which extends the lifespan of infected macrophages, a key viral reservoir. First, we found that HIV-1 infection activates the well characterized pro-survival PI3K/Akt pathway in primary human macrophages, as reflected by decreased PTEN protein expression and increased Akt kinase activity. Interestingly, the expression of HIV-1 or SIV Tat is sufficient to mediate this cytoprotective effect, which is dependent on the basic domain of Tat – a region that has previously been shown to bind p53. Next, we observed that this interaction appears to contribute to the downregulation of PTEN expression, since HIV-1 Tat was found to compete with PTEN for p53 binding; this is known to result in p53 destabilization, with a consequent reduction in PTEN protein production.

**Conclusion:**

Since HIV-1 infected macrophages display highly elevated Akt activity, our results collectively show that PI3K/Akt inhibitors may be a novel therapy for interfering with the establishment of long-living HIV-1 infected reservoirs.

## Introduction

A hallmark of HIV pathogenesis is the loss of CD4+ T cells in HIV-1 infected patients. Infected CD4+ T cells initially undergo cell cycle arrest at G2 caused by a viral accessory protein, Vpr, and eventually cytolysis [1,2]. However, the cell fate and molecular consequences of non-dividing target cells of HIV-1 such as macrophages and microglia are poorly understood. We recently reported that in contrast to HIV-1 infected CD4+ T cells, infection in primary human macrophages and a microglial cell line (CHME5) leads to an extended life span and elevated survival against apoptotic stresses [3]. We also showed that in the HIV-1 transduced CHME-5 microglial cell line, this cytoprotective phenotype is induced by intracellular expression of HIV-1 Tat, which plays a primary role in the transcriptional activation of the HIV-1 LTR [4,5].

HIV-1 infected microglia, brain macrophages, are known to secrete various toxic products such as the Tat and Envelope (Env) proteins, which lead to the death of neighboring neurons and eventually HIV-1 associated dementia (HAD) in the infected host [6-9]. In addition to the secretion of viral proteins, it is known that in the central nervous system (CNS) HIV-1 infected microglia produce nitric oxide (NO), which contributes to the establishment of a highly apoptotic environment in close proximity to infected microglia [10-12]. Even though non-dividing HIV-1 target cells are exposed to these toxic conditions nearby, it has been reported that both microglia and tissue macrophages continue to produce virus for prolonged periods of time. Indeed, a number of studies have suggested that these non-dividing HIV-1 target cells serve as long-living viral reservoirs [13-15].

The PI3K/Akt cell survival pathway has been extensively studied, and has been recognized as a promising target for anti-cancer therapies because its activation is a key cellular event during tumorigenesis [16]. Once PI3K and Akt kinase are activated upon apoptotic stress, they further transduce signals to a series of downstream regulators of cell survival. In its normal state, the PI3K/Akt pathway is negatively regulated by PTEN (phosphatase tensin homolog), which converts PIP3 to PIP2 [17]. We recently observed in our microglial cell line model, that the PI3K inhibitors wortmannin and LY294002 were able to render HIV-1 infected CHME5s susceptible to cell death following an apoptotic stimulus [3].

In this report, we employed primary human macrophages, an important HIV-1 target cell type and viral reservoir, and investigated the specific molecular mechanisms involved in the modulation of the PI3K/Akt pathway. Importantly, we provide virological evidence that supports the application of anti-PI3K/Akt reagents as a potential anti-HIV-1 strategy to eradicate long-living HIV-1 infected human macrophages and to prevent HIV-1 production from these viral reservoirs.

## Results

### PI3K/Akt inhibitors reduce HIV-1 production from infected primary human macrophages

**Figure 1:**
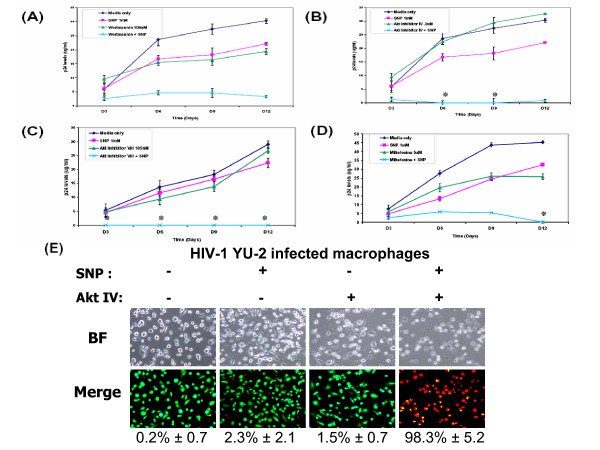
**Treatment of HIV-infected macrophages with PI3K/Akt inhibitors reduces HIV-1 production and induces cell death**. Primary human macrophages were infected with HIV-1 YU-2 and either left untreated (media only) or were treated with one of four different PI3K/Akt kinase inhibitors in the presence or absence of stress (SNP, 1 mM): **(A) **the PI3K inhibitor wortmannin (100 nM), **(B) **Akt inhibitor IV (200 nM), **(C) **Akt inhibitor VIII (105 nM) or **(D) **Miltefosine 5 μM. Viral supernatants were collected every 3 days for 12 days and supernatants were analyzed using the HIV-1 p24 EIA. Asterisks denote undetectable p24 levels. **(E) **On day 12, YU2-infected macrophages were analyzed for cell viability using the live/dead assay. Viable cells are green; dead cells are red. Results are representative of 5 independent, triplicate experiments using cells obtained from multiple blood donors. BF: Bright field. Merge: overlay of red and green fluorescence. The average ± SD percentage of dead cells is also shown.

We previously reported that HIV-1 infection of primary human macrophages and the CHME-5 microglial cell line results in a cytoprotective effect. The prolonged cell survival of HIV-1 infected human macrophages may therefore contribute to the continuous production of HIV-1 progeny from these cells. In an attempt to target the cellular signaling mechanism associated with the increased survival of HIV-1 infected macrophage, we tested whether treatment of HIV-1 infected human macrophages with PI3K/Akt inhibitors could reduce virus production and cell survival. For this test, we employed primary human macrophages and the M-tropic HIV-1 strain, YU-2. First, primary human macrophages were infected with either infectious or heat-inactivated YU-2. To mimic the stressful environment that infected cells are exposed to during HIV-1 infection, human macrophages were treated with SNP, which generates cytotoxic nitric oxide (NO), a compound known to be highly elevated in HIV-infected cells. Three days later, cells were treated with either media alone, SNP alone, a PI3K/Akt inhibitor alone or a mixture of SNP and a PI3K/Akt inhibitor. To inhibit Akt, two commercially available inhibitors, Akt inhibitor IV and VIII (Calbiochem), and a clinically available Akt inhibitor, Miltefosine, approved for treatment of breast cancer were used. In addition, we also employed a broad PI3K inhibitor, wortmannin, for inhibition of the PI3K/Akt pathway. Following treatment as described above, viral production was then monitored for 12 days by p24 ELISA. In order to maintain constant cellular stress, inhibitors and SNP were replenished every 3 days. As shown in Figures 1A–D, SNP treatment alone did not significantly alter viral production as compared to media alone. This indicates that the HIV-1 infected macrophages were able to produce viral particles continuously even after 12 days of constant NO stress. Some studies have actually reported an increase in viral production following treatment with SNP [18,19]. However, this may be a concentration dependent effect since a higher concentration of SNP was used in our experiments to induce a stressful environment. Importantly, however, we did not observe a drastic decrease in viral production following treatment of infected macrophages with SNP alone. In addition, treatment with either wortmannin or the Akt inhibitors alone did not significantly reduce virus production in infected macrophages (Figures 1A–D). However, upon treatment with both SNP and wortmannin, Akt inhibitor IV, Akt inhibitor VIII or Miltefosine (Figure 1A–D), viral production was significantly reduced. Also, as denoted by the asterisks in Figures 1B–D, viral p24 levels were undetectable at various time points post-treatment with both the Akt inhibitor and SNP.

Since the Akt pathway is a well-characterized pathway for cell survival and HIV-1 infected macrophages exhibit an enhanced survival phenotype, we next tested whether the delayed viral production following exposure to Akt inhibitor and stress (SNP) was related to the induction of cell death in HIV-1 infected macrophages. For this test, we quantified cell death under four experimental conditions using the Live/Dead assay (Figure 1E). This assay uses fluorescent dyes which distinguish live cells (green) from dead cells (red) on the basis of intracellular esterase activity (viable) and incorporation of the ethidium homodimer (nonviable). As expected, treatment with either SNP or either of the PI3K/Akt inhibitors alone did not induce significant amounts of cell death in infected macrophages. However, HIV-1 infected macrophages exposed to both SNP and the PI3K/Akt inhibitors clearly displayed a high percentage of cell death (as shown by the extensive red staining in Figure 1E; results for wortmannin, Akt inhibitor VIII and Miltefosine were similar; data not shown). Macrophages treated with heat-inactivated YU-2 underwent high levels of cell death following SNP treatment and combined treatment with inhibitor and SNP, further supporting our observation of an extended survival phenotype in HIV-1 infected macrophages (data shown in previous manuscript, [3]). The percentage of cell death induced under each condition is shown below the panel (Figure 1E). These data suggest that the decrease in viral production is secondary to the induction of cell death, following exposure to PI3K/Akt inhibitors.

### HIV-1 infection reduces PTEN levels in primary human macrophages

**Figure 2:**
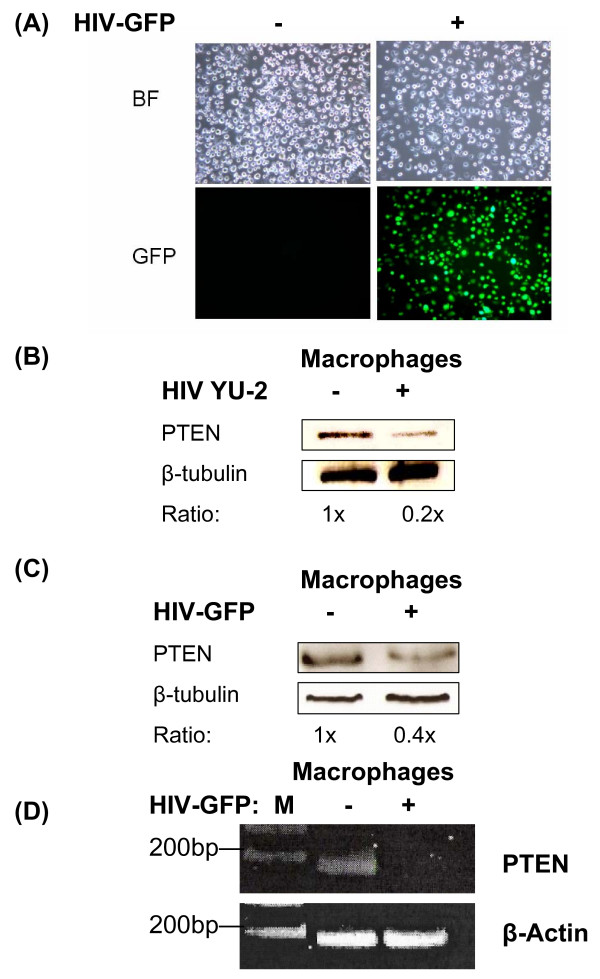
**HIV-1 expression reduces PTEN levels in primary human macrophages**. **(A) **Images of primary macrophages transduced with HIV vector expressing HIV-1 proteins and EGFP (+) or heat-inactivated vector (-). Levels of PTEN protein in YU-2 infected macrophage **(B) **and HIV vector-transduced macrophages **(C) **as determined by Western blotting. Ratios of PTEN normalized by β-tubulin levels are shown. **(D) **Reverse transcriptase PCR analysis of PTEN mRNA levels following transduction of macrophage with HIV vector (+) or treatment with heat-inactivated vector (-). M: 100 bp size marker. β-tubulin and β-Actin were used for loading controls in the Western analysis and RT-PCR, respectively.

Based on the observed potential antiviral activity of the PI3K/Akt inhibitors in primary human macrophages as well as the previous data obtained from the CHME5 cell line [3], we sought to discover the specific molecular mechanisms associated with the cytoprotective effect of HIV-1 infection in human primary macrophages. First, we began to examine various signaling components of this survival pathway to better understand how HIV-1 infected macrophages exhibit prolonged survival and how the inhibition of Akt can lead to the induction of cell death in this viral reservoir. We previously reported that HIV-1 infection leads to extended survival in primary human macrophages and CHME5 microglial cells using both infectious M-tropic HIV-1 YU-2 and an Env and Nef deleted GFP expressing HIV-1 vector (HIV-GFP). Interestingly, CHME5 cells transduced with the HIV-GFP vector, displayed reduced levels of PTEN, a key cellular PI3K/Akt antagonist, compared to CHME5 cells incubated with heat-inactivated vector. It has been described in a number of human cancers that genetic inhibition of PTEN enhances cell survival by facilitating the activation of the PI3K/Akt pathway [17,20-22]. As a result, we hypothesized that PTEN could be targeted by HIV-1 and that interference with PTEN may play a role in the cytoprotective effect exerted in virus-infected macrophages. Therefore, we tested whether HIV-1 infection also reduces the level of PTEN protein in primary human macrophages, which are a key reservoir for HIV-1. Human macrophages were either infected with M-tropic HIV-1 YU-2 (MOI 40) or transduced with HIV-GFP (MOI 40) using heat-inactivated virus or vector as a control. The transduction efficiency was measured by GFP expression (Figure 2A). Cell lysates were prepared 48 hours post-transduction and the level of PTEN protein was measured by Western blotting using β-tubulin as a loading control (Figures 2B and 2C). As shown in Figure 2B, macrophages infected with HIV-1 YU-2 exhibited drastically reduced levels of PTEN protein, displaying approximately 20% of the PTEN level detected in control macrophages. Similarly, HIV-GFP transduced macrophages also exhibited a reduction in PTEN levels to about 40% of the control (Figure 2C), which is very similar to that observed during oncogenic cellular transformation and activation of the PI3K survival pathway [3,23,24].

We also examined PTEN mRNA levels following transduction of our pseudotyped HIV-GFP vector in macrophage by reverse transcriptase PCR (RT-PCR). As shown in Figure 2D, pseudotyped HIV vector-transduced macrophages displayed drastically decreased levels of mRNA compared to the heat-inactivated vector control. The observed decrease in mRNA was more pronounced than the decrease in PTEN protein levels. This is probably because the half life of endogenous PTEN protein is relatively long at about 30 hours [25]. Collectively, these data demonstrate that the cytoprotective effect in macrophages upon infection with HIV-1 YU-2 or transduction with HIV-GFP is likely due to the downregulation of PTEN mRNA and protein levels, which can facilitate the activation of the PI3K/Akt survival pathway.

### HIV-1 infection promotes recruitment of Akt to the plasma membrane via its PH domain and results in increased Akt kinase activity in primary human macrophages

**Figure 3:**
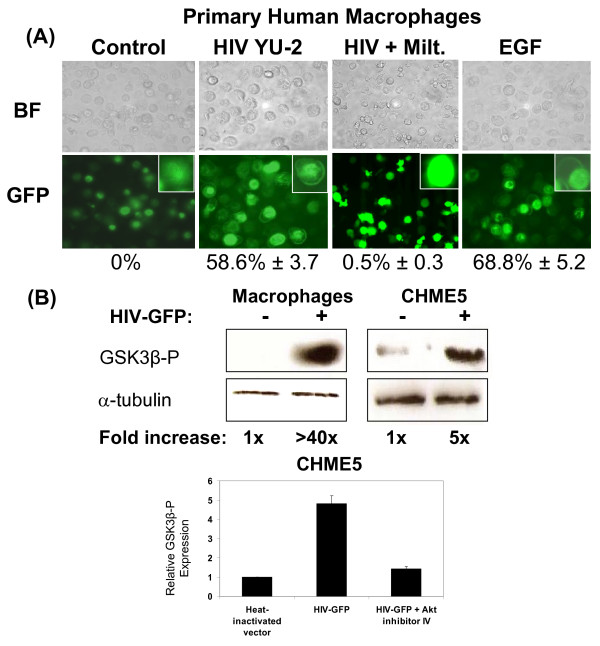
**HIV-1 infection promotes membrane recruitment of Akt's PH domain, resulting in increased Akt activity**. **(A) **Primary human macrophages were sequentially infected with M-tropic HIV-1 YU-2 and Ad.CMV-EGFP-PHAkt expressing the PH domain of Akt, and localization of the PH domain of Akt was assessed by fluorescence microscopy. Heat inactivated YU-2 was used as a negative control, and treatment with epidermal growth factor (EGF) was used as a positive control for Akt activation. HIV-1 infected macrophages were treated with 10 μM Miltefosine (Milt.) for inhibition of Akt. BF: bright field. GFP: green fluorescent protein. Inset: High magnification images of representative cells. The percentage of membrane localized PH-Akt is shown with the SD from three independent experiments. **(B) **Assay for Akt kinase activity. Macrophage and CHME5 cells were transduced with HIV vector and lysed. Using these lysates, an Akt kinase activity assay was performed using GSK3β as a substrate. Western blots of phospho-GSK3β (GSK3β-P) and α-Tubulin (loading control) are shown along with the fold induction of Akt kinase activity relative to control. Fold increase of Akt kinase activity is also shown. The error bars denote the SD from three independent experiments.

The PTEN phosphatase normally converts PIP3 to PIP2. During the activation of the cell survival pathway, high levels of PIP3 lead to the recruitment of the Akt kinase to the plasma membrane by binding to the PH domain of Akt. Therefore, we investigated the effect of HIV-1 infection on the membrane recruitment of Akt. For this, we employed an adenoviral vector that expresses an EGFP-PH fusion protein, in which the PH domain of Akt was fused to the C-terminus of EGFP (Ad.CMV-EGFP-PHAkt). In order to detect the localization of PH Akt during HIV-1 infection, we first infected primary human macrophages with HIV-1 YU-2 and transduced these infected cells 48 hours later with Ad.CMV-EGFP-PHAkt. As shown in Figure 3A, macrophages treated with heat-inactivated HIV-1 displayed diffuse localization of the PH domain throughout the cell. In contrast, HIV-1 YU-2 infection resulted in a distinct localization of EGFP-PHAkt to the plasma membrane. This membrane localization is typically observed following treatment with epidermal growth factor (EGF), which is known to activate the PI3K/Akt pathway [26,27]. Interestingly, we also found that treatment of HIV-1 infected macrophages with the Akt inhibitor Miltefosine inhibited the recruitment of PH-AktGFP to the plasma membrane (Figure 3A). Since Miltefosine inhibits Akt through mimicry of the PH domain, it is likely that Miltefosine binds to PIP3, blocking the recruitment of PH-Akt to the membrane. The percentage of macrophages in which PH domain membrane recruitment was observed is shown below panel 3A. These results suggest that HIV-1 infection in macrophages induces plasma membrane recruitment of Akt which can be reversed using Miltefosine, and our results above suggest that this is likely due to the reduced levels of PTEN expression.

Since plasma membrane recruitment of Akt kinase typically results in increased phosphorylation and activation of Akt, we hypothesized that HIV-1 infection might lead to an increase in Akt kinase activity. Once phosphorylated and activated, the Akt kinase phosphorylates a series of downstream signals including GSK3β [28-31]. To test our hypothesis, we prepared cell lysates from HIV-GFP transduced macrophages and employed an Akt kinase activity assay which uses active Akt kinase from cell lysates to phosphorylate GSK3β substrate. As shown in Figure 3B, macrophages transduced with HIV-GFP displayed an approximately 40-fold increase in Akt kinase activity over the cells treated with heat-inactivated vector.

We also tested Akt activity in the CHME5 cell line (Figure 3B), and similar results were obtained although the increase in kinase activity was substantially less, due to a high level of basal Akt activity. Interestingly, pre-treatment of vector-transduced CHME5 cells with a potent Akt kinase inhibitor, Akt inhibitor IV [32], reduced Akt kinase activity to a basal level similar to that observed in cells treated with heat-inactivated vector (Figure 3B). This confirms that the HIV-1 induced increase in survival of both primary macrophages as well as CHME5 cells is likely a result of increased Akt kinase activity. Importantly, this data also supports that the decrease of HIV-1 production by inhibitor treatment, which was observed in Figure 1, is likely due to induction of cell death via inhibition of the Akt survival pathway

### HIV-1 Tat competes with PTEN for binding to p53

**Figure 4:**
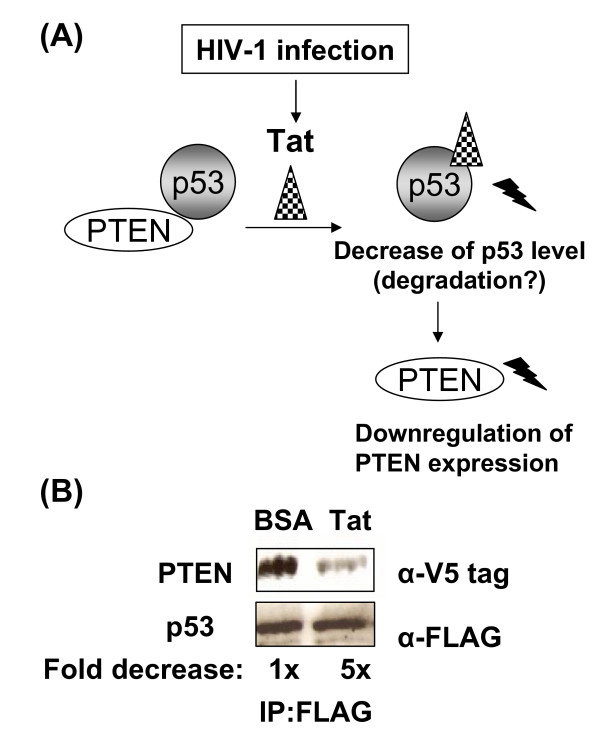
**Binding of HIV-1 Tat to p53 decreases levels of PTEN**. **(A) **Proposed mechanistic circuit for intracellular HIV-1 Tat: HIV-1 Tat may increase cell survival by preventing PTEN from binding to p53. Binding of HIV-1 Tat to p53 may result in reduced levels of both p53 by destabilization and PTEN by downregulation of PTEN expression. **(B) **In vitro binding assay: Lysates containing p53-FLAG were incubated with BSA (control) or HIV-1 Tat protein and then mixed with lysate containing PTEN V5-tag. Proteins bound to p53 were immunoprecipitated using anti-FLAG immobilized antibody and analyzed for PTEN-V5 tag levels by Western blotting. Ratios of PTEN-V5 levels normalized by p53-FLAG levels are shown.

Next, we further tested the molecular mechanisms of the virological factor involved in the HIV-1 induced long-term survival of macrophages. It has been known that HIV-1 Tat protein directly interacts with the C-terminal region of p53 [33,34], but the virological role of this interaction remains speculative. We recently reported that HIV-1 infection and Tat expression leads to the reduction of the transcriptional activator function of p53 [3]. Interestingly, like Tat, PTEN also physically binds to the C-terminal region of p53 and this interaction stabilizes p53 [35]. Since p53 is a key transcriptional activator of PTEN, the stabilization of p53 through PTEN binding enhances the cellular levels of PTEN which in turn leads to repression of the PI3K/Akt pathway in normal cells [17]. Based on these observations, we proposed a possible mechanistic circuit of the cytoprotective effect exerted by HIV-1 infection and intracellular Tat protein (Figure 4A): the decrease in PTEN levels observed during HIV-1 infection and Tat expression may result from the possible destabilization of p53, caused by the direct binding of intracellular Tat to p53. This direct interaction could prevent PTEN from binding to the C-terminal region of p53.

To test this, we performed an in vitro binding assay based on the hypothesized competition between Tat and PTEN for binding to p53 (Figure 4B). p53-containing cell lysates were incubated for a defined length of time with either an irrelevant control protein (BSA) or full-length Tat (101 amino acids) to allow binding. Each lysate was then incubated with normalized amounts of PTEN expressing cell lysates (10 μg). p53 complexes were collected by FLAG-tag immunoprecipitation and examined by Western blot analysis using antibodies directed against the C-terminal V5-tag of PTEN. As seen in Figure 4B, the binding of PTEN to p53 was drastically reduced following incubation with Tat, compared to the BSA control. Instead of using purified tat protein, we could have co-expressed Tat in CHME5 cells. However, since intracellular Tat can decrease p53 levels, this may make it technically difficult to pull down detectable levels of p53. These data support our model circuit in which intracellular Tat prevents PTEN from binding to p53 by interacting with the p53 C-terminal domain. This molecular competition event may facilitate the activation of the PI3K/Akt survival pathway in HIV-1 infected macrophages.

### The Basic domain of Tat is involved in the cytoprotective effect induced by HIV-1 Tat in primary human macrophages

**Figure 6:**
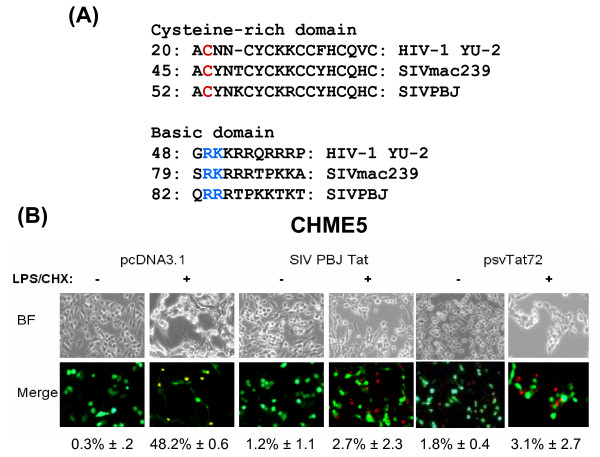
**SIV Tat also exerts a cytoprotective effect**. **(A) **Sequence comparison of the cysteine-rich and basic domains from HIV-1 YU-2, SIV mac239 and SIV PBJ. Numbers indicate residues on the first amino acids of the shown sequences. Colored residues in HIV-1 Tat were mutated in this study. **(B) **CHME5 cells were cotransfected with a plasmid encoding GFP and constructs expressing the first exon of HIV-1 Tat (psvTat72), SIV-PBJ Tat, or with an empty plasmid (pcDNA3.1) using Lipofectamine. Cells were then treated with LPS/CHX and analyzed for cell death. Bright fields (BF) and merged (red+green) fields are shown. Transfected cells are GFP+ cells (green), dead cells (red). The percentage of cell death induced in GFP+, EthD+ cells is shown with the SD from three independent experiments.

Next, we attempted to identify the domain(s) of Tat protein that is/are responsible for the cytoprotective effect. Here, two highly conserved functional domains of Tat protein were investigated: the cysteine rich domain, a domain required for the transactivation activity of HIV-1 Tat [36,37], and the basic domain, which is involved in the cellular uptake, nuclear localization, and transcriptional transactivator functions of Tat [36,38-40] (see Figure 6A for amino acid sequences).

**Figure 5:**
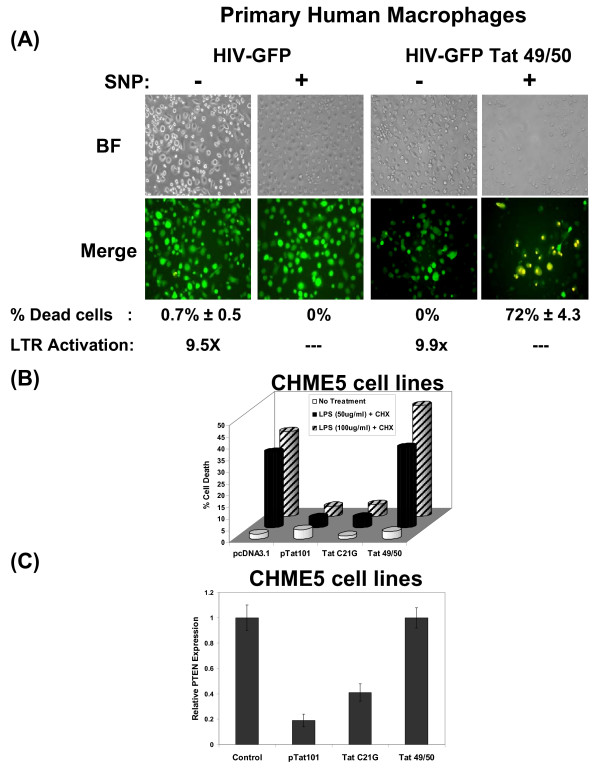
**The Basic domain of Tat is involved in the cytoprotective effect of HIV-1 Tat**. **(A) **Primary human macrophages were transduced with either HIV-GFP wt vector or HIV-GFP Tat 49/50 vector and treated with SNP 1mM for 24 hours. Cell death was then analyzed using a vital dye (red cells = dead). Transduced cells are shown in green (GFP+) while transduced/dead cells are shown in yellow (red+green merge; numbers reflect the % yellow cells in ~200 green cells). The average percentage of cell death and the standard deviation between three independent experiments in triplicate is shown. Luciferase assay results for fold activation of the HIV-1 LTR for the wildtype and the Tat basic domain mutant vectors are also shown. BF: bright field. **(B) **CHME5 sublines expressing wildtype or mutant HIV-1 Tat proteins were exposed to LPS/CHX for 24 hours and analyzed for viability using the trypan blue assay. Results are shown as percent cell death. **(C) **CHME5 sublines expressing wildtype or mutant Tat CHME5 sublines were lysed and analyzed for PTEN protein levels by Western blot. Normalized expression levels of PTEN (relative to α-tubulin) are shown.

Since the interaction between PTEN and p53 seem to be important in the extended survival phenotype and because the binding of Tat to p53 occurs through its basic domain, we first constructed an HIV-GFP vector containing the R49Q/K50E mutations in the basic domain of Tat (HIV-Tat 49/50). The HIV-Tat 49/50 mutant vector was able to transduce macrophages, indicating that the Tat 49/50 mutant still harbors the transcription activator function for activation of the HIV LTR (Figure 5A). Primary human macrophages were transduced with either a wildtype HIV vector or the HIV-Tat 49/50 vector containing the basic domain mutations. For the induction of cell death, transduced macrophages were either left untreated or were treated for 24 hours with sodium nitroprusside (SNP), an NO donor. To monitor cell death we applied the Live/Dead assay, which was described earlier. As shown in Figure 5A, macrophages transduced with either the wildtype or mutant HIV vector alone (without SNP) did not undergo cell death. However, following treatment with SNP, macrophages expressing the Tat 49/50 mutant displayed a greatly increased level of cell death (72%) as shown by the presence of yellow cells (red/green merge) while the wildtype vector-transduced cells exhibited little to no cell death (Figure 5A). Macrophages treated with heat-inactivated vector underwent efficient cell death following SNP treatment as described previously [3]. Therefore, these data confirm that the basic domain of Tat plays a role in the cytoprotective effect exerted by HIV-1 infection and intracellular HIV-1 Tat.

Next, we performed similar experiments using CHME5 cell sublines expressing either wildtype or one of two mutant Tat constructs, the basic domain mutant R49Q/K50E or the transactivation mutant C21G (cysteine-rich domain: see Figure 6A). As expected, the C21G Tat mutant exhibited a defect in transactivator function while the construct harboring the basic domain mutation failed to decrease p53 activity (data not shown). Importantly, the basic domain mutant retained transactivation activity similar to wildtype Tat (data not shown). The alterations in p53 activity following expression of Tat 49/50 could be due to the abrogation of the interaction between p53 and Tat, since this binding is known to occur through the basic domain [33]. Next, we tested the survival ability of the CHME5 sublines (wild type and mutant) by exposing cells to *E. coli *lipopolysaccharide (LPS) and cycloheximide (CHX), and analyzing for the induction of cell death. As shown in Figure 5B, the C21G Tat mutant was still able to exert the cytoprotective effect of wildtype Tat in CHME5 cells while the R49Q/K50E basic domain mutant Tat failed to protect CHME5 cells from the apoptotic stress of LPS/CHX treatment.

We further tested the effect of these Tat mutants on cellular PTEN levels. For this, CHME5 sublines stably transfected with plasmids encoding pcDNA3.1, wild type, C21G or R49Q/K50E Tat protein were analyzed for PTEN expression by Western blotting. Levels of PTEN protein were normalized by α-tubulin protein levels. As shown in Figure 5C, CHME5 cells expressing the R49Q/K50E Tat mutant failed to decrease PTEN protein levels, while CHME5 cells expressing the C21G Tat mutant displayed reduced levels of PTEN similar to wildtype Tat. These data are consistent with the cytoprotective phenotypes of the cells expressing these mutants (Figure 5B). Together, these data suggest that mutations in the Tat basic domain may alter binding of HIV-1 Tat to p53, resulting in increased PTEN levels and consequently an increased incidence of cell death.

### SIV Tat also mediates a cytoprotective effect

SIV and HIV Tat contain a stretch of conserved cysteine residues in the transactivation domain as well as a region rich in basic residues, as shown in the sequence comparison of the cysteine rich and basic domains of HIV-1 (YU-2), SIVmac239 and SIV_PBJ _Tat proteins (Figure 6A). Therefore, we tested whether the expression of SIV Tat could also induce extended survival of CHME5 cells. A plasmid expressing either the first exon of HIV-1 Tat (psvTat72: [41]) or SIV_PBJ _Tat was transfected into CHME5 cells. We also co-transfected a GFP expression plasmid to identify transfected cells expressing Tat. The transfected cells were exposed to LPS/CHX and their survival capability was monitored with the Live/Dead assay. As shown in Figure 6B, CHME5 cells expressing either psvTat72 or SIV_PBJ _Tat (GFP+) displayed enhanced survival as compared to control cells transfected with pcDNA3.1 and GFP (green). The percentage of only the transfected, GFP+ cells undergoing cell death (red) is shown below each panel. These data suggest that the C-terminal region of Tat encoded in exon 2 of the Tat gene is not required for the cytoprotective activity of Tat in CHME5 cells. These results also show that SIV_PBJ _Tat is capable of exerting a cytoprotective effect in CHME5 cells, supporting the possibility that Tat's effects on macrophage/microglial cell survival are conserved among lentiviruses.

## Discussion

**Figure 7:**
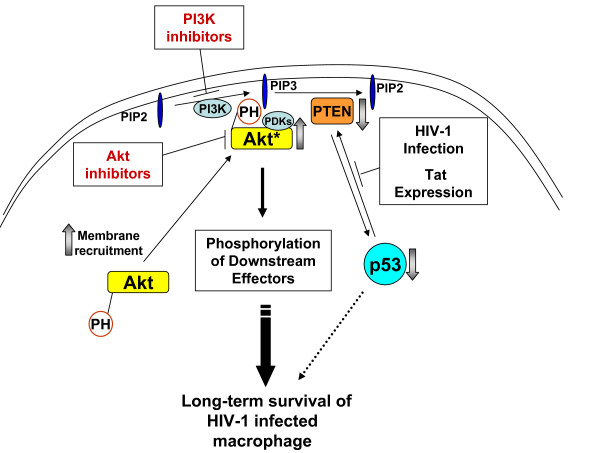
**Mechanistic model for the cytoprotective effect of HIV-1 infection and Tat expression**. A summary of the findings induced by HIV-1 infection and intracellular Tat expression, including the mechanistic actions leading to activation of the PI3K/Akt pathway and subsequent long-term survival of macrophages, is shown. Observed alterations in the signaling pathway induced by HIV-1 infection are shown in block arrows. The dotted arrow indicates an alternative possible cytoprotective effect caused by p53 downregulation. Akt with asterisk denotes the activated/phosphorylated form of the protein.

In this study, we identified PI3K/Akt inhibitors as a novel anti-HIV therapy and examined the specific molecular mechanisms involved in the cytoprotective effect of HIV-1 infection in primary human macrophages. As summarized in Figure 7, our study revealed that HIV-1 expression in macrophages triggers a series of key cellular events typically observed during cell survival activation: PTEN reduction, membrane localization of Akt and elevated Akt kinase activity. Interestingly, treatment of HIV-1 transduced macrophages with the Akt inhibitor Miltefosine was able to reverse the recruitment of PH-Akt to the plasma membrane and the downstream activation of Akt kinase (Figure 3). These cellular alterations, together with the previously reported reduction in p53 activity, mechanistically explain the extended survival phenotype of HIV-1 infected macrophages under stress conditions. This increase in survival of HIV-1 infected macrophages is likely to contribute to viral production and establishment of macrophages as long-lived viral reservoirs.

Mutational studies revealed a novel cytoprotective role for the basic domain of Tat protein. We also observed a decrease in PTEN binding to p53 in the presence of intracellular Tat. Mutations in the basic domain of Tat likely interfere with the ability of HIV-1 Tat to bind p53, allowing stabilization of p53 by PTEN and increased PTEN levels, resulting in abrogation of the cytoprotective phenotype in primary macrophages. In addition, we found that SIV Tat was also capable of protecting CHME5 cells from death. This supports the possibility that Tat's cytoprotective function may be conserved among HIV-1 and SIV Tat proteins, and that these two lentiviruses may share a mechanism for promoting the extended survival of infected macrophages and microglia. Indeed, it is also known that SIV infected macrophages serve as a long-living viral reservoir [42]. Therefore, SIV-infected macaque models may be promising in further developing Akt inhibitors as a novel antiviral therapeutic.

Most importantly, we also examined the ability of PI3K/Akt inhibitors to induce cell death specifically in HIV-1 infected macrophages exposed to SNP stress, which simulates the in vivo local toxic environment. A significant decrease in HIV-1 production from infected macrophages was observed upon combined treatment with SNP and PI3K/Akt inhibitors. This finding suggests that PI3K/Akt inhibitors may have utility as a potential new anti-HIV therapy that is able to specifically target non-dividing HIV-1 target cells such as macrophages, which play important roles in pathogenesis as long-lived HIV-1 reservoirs. Interestingly, infected macrophages treated with SNP or inhibitor alone did not display any signs of cell death or decreased viral production, whereas infected macrophages treated with both SNP and the PI3K/Akt inhibitors underwent cell death with little viral production. This observation indicates that the inhibitory effect of the PI3K/Akt inhibitors on viral production from infected macrophages requires a stressed environment-as may occur in vivo, in association with immune activation and cytokine production [43].

More interestingly, the Akt inhibitor Miltefosine, which has undergone multiple clinical trials and has been approved for treatment of breast cancer in Europe and parasite infections in other countries, was also able to inhibit viral production and cell survival in HIV-1 infected macrophages. In addition, we also found that another Akt inhibitor, Perifosine, which is also currently in clinical trials, was able to decrease viral production and induce cell death in HIV-1 infected macrophages (data not shown).

One interesting question is why HIV-1 infected CD4+ T cells undergo cell death. It is plausible that HIV-1 infection (and Tat expression) may promote cell cycle progression in dividing/activated CD4+ T cells. However, in these infected, dividing CD4+ T cells, due to the strong G2 cell cycle arresting activity of HIV-1 Vpr, further progression through the cell cycle and cell survival may be prevented, resulting in cytolysis [44,45]. Another HIV-1 reservoir cell type is the HIV-1 infected resting memory CD4+ T cell [46,47]. It would be interesting to investigate whether HIV-1 infection also activates the PI3K/Akt pathway in these cells, and if so, whether treatment with PI3K/Akt inhibitors results in elimination of these cells.

In addition to the large number of macrophage/microglia in the toxic environment of the CNS during infection, it has been reported that many of the cells producing HIV-1 in the lymph nodes, spleen and intestine of infected hosts are macrophages [48,49]. These tissue macrophages are also known to persistently produce virus for a long period of time, serving as viral reservoirs. Therefore, it is possible that treatment with Akt inhibitors that are unable to cross the blood brain barrier (BBB) would result in eradication of these infected tissue macrophages. Interestingly, however, it was reported that alkyllysophospholipids such as Miltefosine are able to penetrate the BBB [50-53], which supports the potential use of Miltefosine to eradicate viral reservoirs of the CNS.

## Conclusion

This study elucidates the molecular and cellular mechanisms involved in the cytoprotective effect of HIV-1 infection in primary human macrophages and indicates the PI3K/Akt pathway as a key contributor to this effect. It is increasingly apparent that many PI3K/Akt inhibitors under development as anti-cancer therapy are safe and well-tolerated in both experimental animals and humans [54-57]. Indeed, several inhibitors including Miltefosine have been approved for treatment of human cancers. This further supports the possible use of PI3K/Akt inhibitors for anti-HIV therapy and targeting of long-lived viral reservoirs.

## Methods

### Cells, viruses, HIV-1 vectors and plasmids

Primary human monocyte-derived macrophages were isolated from human buffy coats and differentiated as previously described [58]. The CHME5 microglial cell line was maintained as described previously [3]. M-tropic HIV-1 YU-2 was prepared using human PBMCs [3], and VSV-G pseudotyped HIV-1 vectors expressing EGFP and all HIV proteins except Nef and Env were prepared as described [58] and used to transduce primary human macrophages. Vector titers were determined using CHME5 cells, and the p24 EIA was performed for each vector or virus preparation following manufacturer's protocol (PerkinElmer). The plasmid encoding the first exon of Tat, psvTat72, was obtained from the NIH AIDS reagent program. The p53-FLAG plasmid constructed by Dr. Thomas Roberts [59] was purchased from Addgene (plasmid 10838). A plasmid encoding the PTEN gene was a generous gift from Dr. Jim Miller (University of Rochester). Using this plasmid, a linker sequence followed by the V5 tag sequence was introduced by PCR. After construction of PTEN-V5 tag, the tagged gene was inserted into pcDNA3.1+Hygro (Invitrogen) using the *Kpn*I and *Xho*I restriction sites.

### EGFP-PHAkt expressing adenovirus vector

The EGFP-PHAkt fusion gene from pEGFP-PHAkt [60] was cloned into pShuttle-CMV prior to recombination into pAdEasy (Stratagene). Recombinant adenoviral stocks (Ad.CMV-EGFP-PHAkt) were then generated following transfection in HEK293A cells using methods provided by the supplier (Stratagene). The virus was purified by CsCl density gradient centrifugation and the viral titer was determined by real-time PCR on a BioRad icycler (Hercules, CA) using a Taqman probe and primers that amplified a small portion of the Adenovirus hexon gene [61].

### PHAkt membrane localization

Primary human macrophages (5 × 10^4 ^cells) were infected with HIV-1 YU-2 (MOI = 40) for 48 hours. Heat-inactivated YU-2 was used as a control. Cells were washed with DPBS and transduced with Ad.CMV-EGFP-PHAkt (MOI = 3000) for 24 hours. Positive control cells were treated with epidermal growth factor (EGF, Sigma) for 15 minutes before fixation with 3% formaldehyde. For inhibition of membrane localization, infected macrophages were treated with Miltefosine (10 μM) 24 hours post-infection for 48 hours prior to fixation. Macrophages were visualized for the localization of the PH domain of Akt by examining GFP fluorescence on a Leica microscope (200×).

### Akt kinase activity assay

Primary human macrophages (1 × 10^6^) and CHME5 (1 × 10^6^) cells were transduced with pseudotyped HIV-GFP vector (MOI of 40 for macrophages and MOI of 1 for CHME5 cells), giving ~95% transduction. CHME5 cells were treated with Akt inhibitor IV (.2 μM) for 24 hours following transduction with the HIV-GFP vector where specified. 48 hours post-transduction, cells were lysed using ELB lysis buffer before performing the Akt kinase activity assay (Cell Signaling) as per the manufacturer's protocol. Following incubation with the GSK3β fusion protein, 6 × SDS stop buffer was added and samples were loaded onto an SDS 8% (w/v) polyacrylamide gel and then transferred to nitrocellulose membrane (Hybond, Amersham Biosciences). Using the antibodies supplied, GSK3β-P levels were detected by Western blot analysis. Protein levels were normalized using β-tubulin as a loading control. Each assay was performed in triplicate.

### Reverse transcriptase PCR

Macrophages were transduced with either the HIV-GFP vector or an adenoviral vector expressing GFP +/- Tat. 24 hours post adenoviral transduction or 5 days post HIV vector transduction, cells were lysed for RNA isolation. cDNA was then synthesized from the RNA samples using the Qiagen cDNA synthesis kit (Qiagen, CA) as per the manufacturer's protocol. RT-PCR was then performed using the following primers for PTEN: F primer – 5' TTTGAAGACCATAACCCACCA 3'; R primer – 5' CCATAGAAATCTAGGGCCTCT 3'. The β-actin RT-PCR was performed with the primers as previously described [62].

### Western blotting

Cell lysates were prepared in ELB buffer supplemented with protease inhibitors (Sigma) and phosphatase inhibitor cocktail (Sigma) and samples (10–20 μg) were applied to an SDS 8% (w/v) polyacrylamide gel. The expression of the proteins of interest was detected by probing with the PTEN (138G6) rabbit monoclonal antibody (Cell Signaling) or M2 FLAG mouse antibody (Sigma, 1:1000). Donkey anti-rabbit Ig or sheep anti-mouse Ig (Amersham Biosciences, 1:5000) for secondary antibody followed by ECL detection using the SuperSignal West Femto kit (Pierce). For a loading control, blots were probed for α-tubulin (Cell Signaling) followed by sheep anti-mouse IgG (Amersham Biosciences). Expression of the protein of interest in each sample was normalized to either β-tubulin or p53-FLAG levels for analysis using ImageJ software (NIH), and ratios were determined from experiments in triplicate.

### p53 binding competition assay

CHME5 cells were transfected with either a plasmid encoding p53-FLAG or PTEN-V5 tag. After 24 hours, cells were lysed in ELB buffer. Following normalization of protein concentration of each lysate, p53-FLAG-containing lysate (10 μg) was incubated with Tat101 protein (Xeptagen, 2 μg/ml) and allowed to bind for 30 minutes at 4°C on a rocking platform. Following this incubation, an equal protein amount (10 μg) of PTEN-V5 lysate was added and allowed to bind for an additional 30 minutes at 4°C. The combined lysates were then applied to EZview Red anti-FLAG M2 affinity gel (Sigma) for immunoprecipitation as per the manufacturer's protocol. After elution of the p53 complexes with 6× SDS buffer, lysates were applied to an SDS 8% (w/v) polyacrylamide gel and Western analysis was performed as described above. PTEN expression was detected using a mouse anti-V5 tag antibody (Serotec, 1:1000) followed by sheep anti-mouse IgG (Amersham Biosciences, 1:5000). ECL detection was then performed as described above and the membrane was reprobed for p53-FLAG as a loading control. Each binding ratio was performed in triplicate.

### Cell death assays

Sodium nitroprusside (Sigma) was used at 1 mM for treatment of macrophages. The live/dead assay was then performed as previously described [3] and percent cell death is shown. For analysis of induction of cell death in the CHME5 Tat and control sublines, cells were exposed to CHX (10 μg/ml) and *E*. coli serotype O26:B6 LPS (Sigma) at a concentration of 50 μg/ml or 100 μg/ml for 24 hours. For the live/dead assay, cells were cotransfected with a plasmid encoding GFP and a construct expressing either SIV_PBJ _Tat or psvTat72 (NIH AIDS Reagent Program). Following treatment with LPS (50 μg/ml) and CHX (10 μg/ml), cells were analyzed for cell death using the Live/Dead Cytotoxicity/Viability assay (Molecular Probes) as per the manufacturer's protocol. Images were taken at a magnification of 200 × 24 h post-treatment using a fluorescence microscope (Leica). Each assay was performed in triplicate.

### Construction of HIV-1 Tat mutants

The control (pcDNA3.1) and wildtype Tat (pTat101) expressing CHME5 sublines were constructed as previously described [3]. For creation of the transactivation mutant C21G, the sequence at amino acids 20/21 was changed to GCCGGC by site-directed mutagenesis while amino acids 49 and 50 (Arg and Lys) were changed to CAGGAG (Glu and Gln) to create the basic domain mutant. The C21G and 49/50 Tat mutants were then cloned into the pcDNA3.1+Hygro plasmid (Invitrogen). To create stably expressing CHME5 sublines, cells were transfected with the above constructs and selected using hygromycin for two weeks. The resulting CHME5 sublines were referred to as C21G (transactivation mutant) and Tat 49/50 (basic domain mutant). Three independent subline clones were isolated and used for characterization. The cell lines were tested for transactivation activity using a luciferase assay measuring activation of the HIV-1 LTR. A plasmid encoding the HIV-1 LTR promoter region fused to a luciferase cassette was transfected into the CHME5 cell lines and 24 hours later, cells were lysed and the luciferase assay was performed as previously described [3].

In order to construct the pseudotyped vector system expressing the Tat 49/50 mutant, the Quickchange II XL kit (Stratagene) was used. The following primers were used to target amino acids 49 and 50 of the Tat gene, changing the AGGAAG sequence to CAGGAG: F primer – 5' GGCATCTCCTATGGCCAGG AGAAGCGGAGACAGCG 3'; R primer – 5' CGCTGTCTCCGCTTCTCCTGG CCATAGGAGATGCC 3'. The sequence of the vector was confirmed by sequencing.

### Virus production assay

Primary human macrophages were infected with HIV-1 YU-2 (1 × 10^5 ^pg). Three days post-infection, cells were washed with DPBS to eliminate the presence of virus. After washing, cells were cultured either in media alone, media containing SNP (1 mM) only, media supplemented with the PI3K inhibitor wortmannin (100 nM, Sigma) or the specific Akt inhibitors IV, VIII (200 nM, 105 nM, Calbiochem) [32] or Miltefosine (5 uM, Cayman Chemicals) only or a combination of inhibitors and SNP. Infected macrophages were cultured for 12 days, during which time viral supernatants were collected and fresh media with inhibitors or SNP was also added every 3 days. The p24 levels contained in each viral supernatant sample at the various time points was monitored using the p24 enzyme-linked immunosorbent assay (Beckman-Coulter) according to the manufacturer's protocol. Viral production was plotted over time and asterisks denote undetectable p24 levels. On day 12, cells were analyzed for the induction of cell death using the live/dead assay as described above. Bright fields and merged (red + green) fields are shown. Green cells represent viable cells while red-stained cells correspond to dead or dying cells. The average percentage of cell death induced was determined from three independent experiments and is shown with the SD.

## Competing interests

The author(s) declare that they have no competing interests.

## Authors' contributions

PC carried out the studies and drafted the manuscript. BBT constructed and produced the adenoviral vector used in the PH domain localization study. CMRMF constructed the pseudotyped vector encoding the basic domain Tat mutant. VP, SBM and SD helped with interpretation of data and revision of the manuscript as well as preparation of reagents. BK conceived of the study, participated in its design and coordination and drafted the manuscript. All authors read and approved the final manuscript.
